# Status of newborn screening and follow up investigations for Mucopolysaccharidoses I and II in Taiwan

**DOI:** 10.1186/s13023-018-0816-4

**Published:** 2018-05-25

**Authors:** Chih-Kuang Chuang, Hsiang-Yu Lin, Tuan-Jen Wang, You-Hsin Huang, Min-Ju Chan, Hsuan-Chieh Liao, Yun-Ting Lo, Li-Yun Wang, Ru-Yi Tu, Yi-Ya Fang, Tzu-Lin Chen, Hui-Chen Ho, Chuan-Chi Chiang, Shuan-Pei Lin

**Affiliations:** 1 Division of Genetics and Metabolism, Department of Medical Research, MacKay Memorial Hospital, Taipei, Taiwan; 2 Department of Pediatrics, MacKay Memorial Hospital, Taipei, Taiwan; 3 Department of Laboratory Medicine, MacKay Memorial Hospital, Taipei, Taiwan; 4 The Rare Disease Center, MacKay Memorial Hospital, Taipei, Taiwan; 5 The Chinese Foundation of Health, Neonatal Screening Center, Taipei, Taiwan; 6 Taipei Institute of Pathology, Neonatal Screening Center, Taipei, Taiwan; 7 College of Medicine, Fu-Jen Catholic University, Taipei, Taiwan; 8 Department of Early Childhood Care and Education, Mackay Junior College of Medicine, Nursing and Management, Taipei, Taiwan; 9 Department of Medicine, MacKay Medical College, Taipei, Taiwan; 10 Department of Medical Research, China Medical University Hospital, China Medical University, Taichung, Taiwan; 11 Department of Infant and Child Care, National Taipei University of Nursing and Health Sciences, Taipei, Taiwan; 12 Departments of Pediatrics and Medical Research, MacKay Memorial Hospital, No. 92, Sec. 2, Chung-Shan N. Rd, Taipei, 10449 Taiwan

**Keywords:** Mucopolysaccharidoses, Glycosaminoglycans, Tandem mass spectrometry, α-iduronidase, Iduronate-2-sulfatase, DNA sequencing analysis, MPS newborn screening

## Abstract

**Background:**

Mucopolysaccharidoses (MPS) are lysosomal storage diseases in which mutations of genes encoding for lysosomal enzymes cause defects in the degradation of glycosaminoglycans (GAGs). The accumulation of GAGs in lysosomes results in cellular dysfunction and clinical abnormalities. The early initiation of enzyme replacement therapy (ERT) can slow or prevent the development of severe clinical manifestations. MPS I and II newborn screening has been available in Taiwan since August 2015. Infants who failed the recheck at recall were referred to MacKay Memorial Hospital for a detailed confirmatory diagnosis.

**Methods:**

From August 2015 to November 2017, 294,196 and 153,032 infants were screened using tandem mass spectrometry for MPS I and MPS II, respectively. Of these infants, 84 suspected cases (eight for MPS I; 76 for MPS II) were referred for confirmation. Urinary first-line biochemistry examinations were performed first, including urinary GAG quantification, two-dimensional electrophoresis, and tandem mass spectrometry assay for predominant disaccharides derived from GAGs. If the results were positive, a confirmative diagnosis was made according to the results of leukocyte enzymatic assay and molecular DNA analysis. Leukocyte pellets were isolated from EDTA blood and used for fluorescent α-iduronidase (IDUA) or iduronate-2-sulfatase (IDS) enzymatic assay. DNA sequencing analysis was also performed.

**Results:**

Normal IDS and IDUA enzyme activities were found in most of the referred cases except for four who were strongly suspected of having MPS I and three who were strongly suspected of having MPS II. Of these infants, three with novel mutations of the IDS gene (c.817C > T, c.1025A > G, and c.311A > T) and four with two missense mutations of the IDUA gene (C.300-3C > G, c.1874A > C; c.1037 T > G, c.1091C > T) showed significant deficiencies in IDS and IDUA enzyme activities (< 5% of mean normal activity), respectively. Urinary dermatan sulfate and heparan sulfate quantitative analyses by tandem mass spectrometry also demonstrated significant elevations. The prevalence rates of MPS I and MPS II in Taiwan were 1.35 and 1.96 per 100,000 live births, respectively.

**Conclusions:**

The early initiation of ERT for MPS can result in better clinical outcomes. An early confirmatory diagnosis increases the probability of receiving appropriate medical care such as ERT quickly enough to avoid irreversible manifestations. All high risk infants identified in this study so far remain asymptomatic and are presumed to be affected with the attenuated disease variants.

## Strengths and limitations of this study

- Newborn screening for Mucopolysaccharidoses I and II has been available in Taiwan since August and October 2015, respectively.
- Infants with suspected MPS were referred to MacKay Memorial Hospital for MPS type confirmation by leukocyte enzymatic assay and molecular DNA analysis.
- Four and three infants with the attenuated variants of MPS I and MPS II respectively were identified; all remain asymptomatic to date.
- The prevalence rates of MPS I and MPS II in Taiwan were 1.35 and 1.96 per 100,000 live births, respectively.

## Background

Mucopolysaccharidoses (MPS), a group of rare genetic diseases known as lysosomal storage disorders (LSDs), are caused by the deficiency of enzymes that catalyze the stepwise degradation of glycosaminoglycans (GAGs). Mucopolysaccharidosis I (MPS I; Hurler, Hurler-Scheie, Scheie syndrome) and MPS II (Hunter syndrome) are diseases characterized by deficiencies in the enzymes α-iduronidase (IDUA; EC 3.2.1.76) and iduronate-2-sulfatase (IDS), respectively. A deficiency in the activity of either one of these enzymes can lead to excessive lysosomal storage of dermatan sulfate (DS) and heparan sulfate (HS) resulting in devastating manifestations such as coarse facial features, developmental delay and decline, gibbus, hepatosplenomegaly, cardiac valve disease, umbilical and inguinal hernias, joint deformity with a restricted range of motion, airway dysfunction with complications, sleep apnea, recurrent otitis media, and premature death [1–4]. Initial symptoms often present during the first 5 years of life depending on the severity of disease and age at diagnosis. MPS I is differentiated as MPS I and MPS I attenuated. MPS II phenotypes are defined as attenuated and severe forms according to their clinical severity [1–4]. All types of MPS are autosomal recessive disorders except for MPS II, which is X-linked, and the defective gene is transmitted from the mother to son.

Few studies have investigated the incidence (or prevalence) of MPS. The overall birth incidence for all patients with MPS was reported to be 2.04 per 100,000 live births in Taiwan [5]. Of these cases, MPS II had the highest birth incidence of 1.07 per 100,000 live births (2.05 per 100,000 male live births), comprising 52% of all diagnosed MPS cases. In addition, the birth incidence rate of MPS I was 0.11 per 100,000 live births, accounting for 6% of all cases of MPS [5]. Compared to the rates reported in different populations, the overall incidence rates of MPS in European countries are very similar, ranging from 1.75 (Sweden) to 4.5 (the Netherlands) per 100,000 live births [6–8].

Recently, several experimental and approved treatments have been reported for MPS subtypes, including hematopoietic stem cell transplantation [9, 10], enzyme replacement therapy (ERT) [11–13], premature stop codon read-through [14, 15], and vector-mediated gene therapy [16–18]. ERTs are available for MPS I, MPS II, MPS IVA and MPS VI. However, the optimal benefits from ERT, particularly for patients with MPS who suffer from devastating soft tissue storage and skeletal diseases with or without central nervous system (CNS) involvement, require commencing treatment before the onset of irreversible clinical disease. In general, the earlier the initiation of ERT for MPS the better the clinical outcomes. One previous study reported notable scoliosis in an affected female child, whereas her little brother received ERT just after birth and appeared to be normal [19]. In addition, another study reported that the early initiation of laronidase treatment prior to the onset of symptoms in patients with attenuated MPS I could slow or prevent the development of severe clinical manifestations [20]. With the exception of cases where there is a family history of the disease, the pre-symptomatic detection of MPS can only be achieved by newborn screening (NBS). Recent progress in newborn screening programs for LSDs has shown promise for their early detection [21–23].

MPS is diagnosed by a decrease in or loss of enzyme activity, usually involving a fluorescent-tagged artificial substrate such as 4*-*methylumbelliferone (4MU) or a natural substrate in which a fragment of biological substrate is labeled with a radio-stable isotope [24–26]. Homogenates of cultured fibroblasts and leukocytes have been widely used to make a definitive diagnosis of MPS. The use of dried blood filter paper samples offers several advantages in terms of cost, transportation and suitability of sample collection from neonates. We previously reported findings from a pilot study of newborn screening for MPS I in Taiwanese infants using the 4MU fluorescent enzymatic assay in which we analyzed more than 35,285 samples from 2008 to 2013. According to the results of that study, the incidence of MPS I in Taiwan was about 1/17,643 [21], which is very close to that (1:14567) reported in a 2015 Missouri pilot study which used a multiplexed fluorometric enzymatic assay (digital microfluidics assay) [27]. Recently, the usage of tandem mass spectrometry to screen multiple LSDs has been established and been shown to be feasible for newborn screening purposes including MPS I. Another two representative and large-scale NBS programs for multiplexed LSDs have been conducted in Illinois and Kentucky since 2014 and 2016, respectively [28, 29], and reported that the incidence and detection rates of MPS I were 1 in 219,793 and 1 in 55,161, respectively. In addition, the tandem mass spectrometry assay for MPS II NBS has been established and extensively used due to a more stable IDS substrate being available [30–33]. An early diagnosis can be achieved by NBS, and MPS I and II NBS programs have been available nationwide at three NBS centers in Taiwan since August 2015. Tandem mass spectrometry for the direct assay of lysosomal enzymes including IDUA and IDS in dried blood spots (DBS) is used in these screening programs. From August 1, 2015 to November 30, 2017, a total of 294,196 and 153,032 infants were analyzed by tandem mass spectrometry assay for MPS I and MPS II, respectively. All DBS specimens were collected with informed parental consent. An abnormal NBS result is first dealt with by repeating the NBS test before additional diagnostic studies are conducted. If the result of the preliminary analysis is positive, it means that the enzyme activity of either IDUA or IDS in DBS is reduced and usually lower than the cut-off value of the first test, and the retest is performed using the same initial DBS sample. The cut-off value of the second test is lower than that of the first test in order to exclude the possibility of false positive cases. If the infants do not pass the retest, the parents of high-risk infants receive a recall notice for the collection of a second DBS sample for tandem mass spectrometry retesting. If the result of the second DBS is positive, the case is considered to be high risk and is referred to genetic referral centers for further evaluation, confirmatory testing and diagnosis. The reasons for the additional collection of a repeat NBS specimen and retesting are carefully explained to the infant’s parents if the initial result of NBS for MPS I or II is positive. Of these infants, 84 suspected cases including eight for MPS I and 76 for MPS II were referred to MacKay Memorial Hospital for further confirmation. The cut-off values were set at < 3.0 μmol/L/H for the first and second tests for MPS I, and < 6.5 and < 2.2 μmol/L/H for MPS II, respectively [32]. The aim of this study was to investigate the current status of several genotypes that may cause pseudo deficiencies in IDS enzyme activity, and also to report the positive findings of MPS I and MPS II through confirmatory diagnostic experiments.

## Methods

**Fig. 1:**
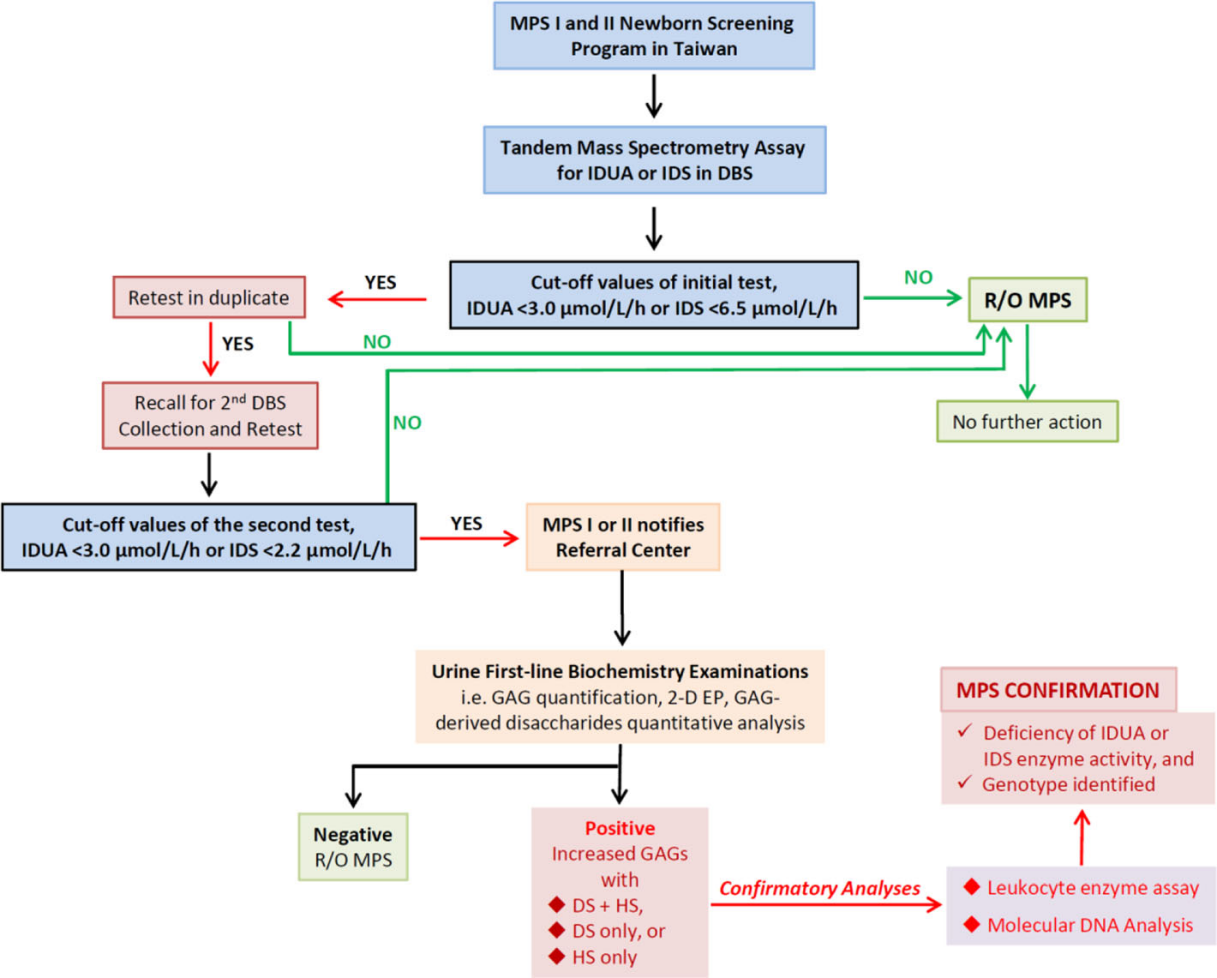
The algorithm of MPS diagnosis. The suspected cases with reductions in enzyme activity in DBSs detected by tandem mass spectrometry were referred to Mackay Memorial Hospital for further confirmation. The cut-off values were 3.0 μmol/L/h for the initial and second DBS for MPS I. For MPS II, the cut-off values were 6.5 μmol/L/h for the initial and 2.2 μmol/L/h for the second DBS. If the result was far below the cut-off value, the case was highly suspected of having MPS, and the recall process was started by the genetic counselor

The algorithm of an MPS diagnosis is illustrated in Fig. 1. Suspected cases with a reduction in enzyme activity in DBS detected by tandem mass spectrometry were referred to Mackay Memorial Hospital for further confirmation. The aim of the confirmatory testing was to rule out the possibility of an MPS and to provide an accurate diagnosis. The cut-off values were 3.0 μmol/L/h for the initial and second DBS for MPS I, and 6.5 μmol/L/h for the initial and 2.2 μmol/L/h for the second DBS for MPS II. If the results were far below the cut-off values, the case was defined as being highly suspected, and the recall process was carried out by a genetic counselor with the arrangement of an outpatient department (OPD) visit and specimen collection. For a laboratory diagnosis, urinary first-line biochemistry examinations were performed first, including GAG quantification, two-dimensional electrophoresis (2-D EP), and the quantitative analysis of relevant GAG-derived disaccharides using tandem mass spectrometry such as chondroitin sulfate (CS), DS, and HS. If the results of the first-line biochemistry examinations were negative, the possibility of MPS could confidently be ruled out. However, if the results were positive, for instance, an increased quantity of GAGs with either DS + HS, or DS only, or HS only, the case was highly suspected of having MPS. Confirmative diagnoses also included leukocyte enzymatic assay and molecular analysis.

The tandem mass spectrometry methods used for MPS I and II NBS have been reported previously [30–33]. The tandem mass spectrometry assay is used due to several advantages, including high through-put, high specificity, high sensitivity, prompt measurement, simple experimental protocol, and the availability of high quality commercial substrates. At present, MPS I and II NBS services are available nationwide at three Newborn Screening Centers in Taiwan. Written consent was obtained from the parents for screening of their newborn for LSDs.

### Samples for confirmation

Suspected cases with a reduction in either IDUA or IDS enzyme activity in DBS detected by tandem mass spectrometry assay were referred to MacKay Memorial Hospital for MPS confirmation after the first and repeat NBS tests. A total of 84 suspected cases including eight for MPS I and 76 for MPS II were analyzed for further confirmation in this study. The samples required for the assay included urine (10-20 mL) and EDTA blood (2 tubes, 3-5 mL in each). Urine samples were stored at − 20 °C prior to GAG analysis, and the blood samples were kept at room temperature and 4 °C before leukocyte isolation for enzymatic assay and molecular DNA analysis, respectively.

### Urinary quantitative GAG analysis

#### The dimethylmethylene blue method (DMB/creatinine ratio)

GAGs were determined quantitatively in urine in a reaction with the dye dimethylmethylene blue (DMB) that did not require prior precipitation of the GAGs. The color was measured rapidly at a wavelength of 520 nm. The DMB ratio was obtained by dividing urine creatinine by the volume of GAGs in mg/L, and the ratio was expressed as mg/mmol creatinine [34, 35]. The quantitative DMB method can give a ratio of excretion of GAGs relative to creatinine, which is age-dependent. A high ratio relevant to age indicates the possibility of having MPS. The urine creatinine level is proportional to age but inversely proportional to the DMB/CRE ratio. A higher DMB/CRE ratio was due mostly to the very young age group (< 6 months), whereas it was lower and nearly constant in the adult group. The normal reference values for the infants in this study were 13.6-66.1 mg/mmol creatinine (39.9±13.1) (< 6 months) and 0-55.2 mg/mmol creatinine (23.3±15.9) (0.6-2 years).

#### Two-dimensional electrophoresis

GAGs were precipitated from urine using Alcian blue (AB) containing sodium acetate. Sodium chloride and methanol were used to dissolve MPS-AB complexes. Sodium carbonate was then sequentially added to dissociate the MPS complex and AB. Finally, ethanol was used to re-precipitate MPS. After drying, the samples were ready for 2-D EP to separate them into component GAGs on cellulose acetate sheets. Electrophoresis was carried out in pyridine-acetic acid buffer in the first direction and barium acetate buffer in the second. The GAGs were visualized by staining with Alcian blue [34].

#### Liquid chromatography/tandem mass spectrometry (LC-MS/MS) to detect predominant GAG-derived disaccharides in urine

LC-MS/MS for the relevant GAG-derived disaccharides, i.e. CS, DS and HS, was performed using methanolysis as described previously [36–38]. The GAGs were precipitated and then degraded to uronic acid-*N*-acetylhexosamine dimers. Methanolysis was performed by adding 3 N HCl in methanol (200 μL). The methyl group (CH_3_) would bind to the COO^−^ (carboxyl group) of C6 of L-iduronate and to the C1 negative oxide ion of N-acetyl-galactosamine-4-sulfate. One particular disaccharide for each GAG was selected. The m/z (mass to charge) of the parent ion and its daughter ion after collision was 426.1 → 236.2 for DS and 384.2 → 161.9 for HS. The internal standards including [^2^H_6_] DS, [^2^H_6_] CS, and [^2^H_6_] HS were prepared in-house by deuteriomethanolysis of GAG standards (CS, DS, and HS) [38]. The mass spectrum of the deuteriomethanolysis products of DS and HS showed the incorporation of + 6 atoms of deuterium. Therefore, the m/z was 432.0 → 239.0 for [^2^H_6_] DS and 390.4 → 162.5 for the [^2^H_6_] HS dimer. Tandem mass spectrometry assay for urinary GAG-derived disaccharides is an excellent approach that can be used to follow up NBS results for possible MPS I and MPS II.

### Leukocyte enzyme assay for MPS I and MPS II

Differential diagnoses of MPS can be achieved using an enzymatic assay [39, 40]. Leukocyte isolation and protein determination are required prior to performing the enzyme assay. Leukocytes were isolated from EDTA blood by centrifugation through Ficoll-Paque (Sigma-Aldrich, Inc., St. Louis, MO, USA) at 18 °C for 40 min at 2500 rpm. By removing the upper layer, the white cell ring from the interface was removed and transferred to a 5 mL centrifuge tube, followed by the addition of 0.9% NaCl to the top, mixing, and centrifugation for 10 min at 2000 rpm at 4 °C. Cell lysates were prepared by suspending leukocyte pellets in 0.2 mL of 0.85% NaCl and disrupted by six cycles of freeze-thawing. Proteins were determined using Coomassie Plus protein assay (Pierce, Thermo Fisher Scientific Inc., Waltham, MA, USA). The assay for individual enzyme activity was performed using 4-methylumbelliferyl substrate. Enzyme activity was proportional to the amount of liberated fluorescence detected (μmol enzyme activities/g protein/hour*).* Individual enzyme activity which was 5% lower than normal was defined as a marked reduction in that enzyme activity.

#### Leukocyte β-iduronidase (IDUA; MPS I)

**Fig. 2:**
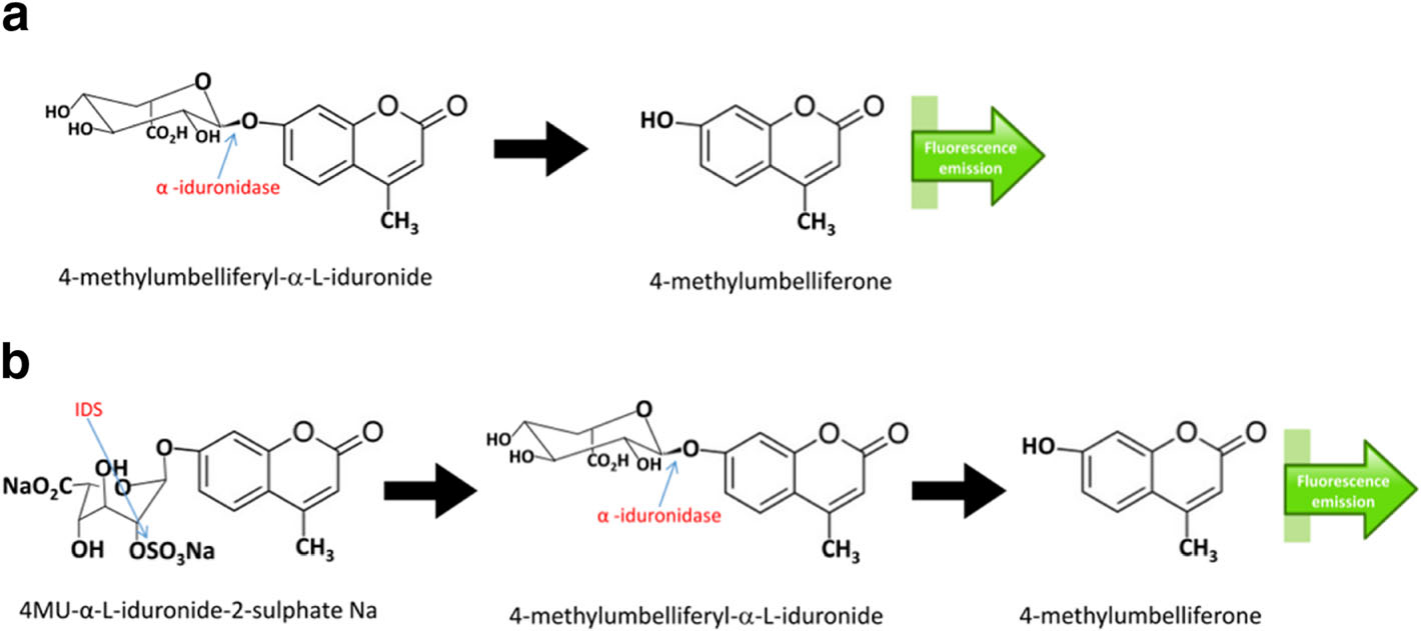
**a** The method utilizes 4-methylumbelliferyl-α-L-iduronide substrate that is hydrolyzed by IDUA into a highly fluorescent product, 4-methylumbelliferone (4MU). The rate of fluorescence increase is directly proportional to enzyme activity. **b** The enzymatic liberation of the fluorochrome from 4MU-α-L-iduronide-2sulfate requires the sequential action of IDS and α-iduronidase

The principle of the IDUA enzymatic assay is illustrated in Fig. 2 (A). Hydrolysis of the synthetic substrate 4-methylumbelliferyl-α-L-iduronide at acidic pH was followed by measuring the fluorescence of the liberated 4MU after stopping the reaction with alkaline buffer. The fluorescence produced was measured using a fluorimeter (Luminescence Spectrometer, Perkin Elmer LS 30, USA). The excitation wavelength was set at 365 nm with an emission of 450 nm. The method used 4-methylumbelliferyl-α-L-iduronide substrate that was hydrolyzed by IDUA into the highly fluorescent product 4MU. The rate of fluorescence increase was directly proportional to enzyme activity [39].

### Leukocyte iduronate-2-sulfate sulfatase (IDS; MPS II)

The principle of the assay is illustrated in Fig. 2b. The enzymatic liberation of fluorochrome from 4MU-α-L-iduronide-2-sulfate requires the sequential action of IDS and α-iduronidase. A normal level of α-iduronidase activity was insufficient to complete the hydrolysis of the reaction intermediate 4-methylumbelliferyl-α-iduronide formed by IDS [40].

### Molecular DNA analysis

Genomic DNA was prepared from peripheral blood leukocytes by high-salt extraction. Polymerase chain reactions (PCRs) of exons found in individual MPS types including adjacent intronic regions were performed with various primers and conditions. PCR amplification of cDNA or genomic DNA in patients and unaffected controls was carried out using oligonucleotide primers, i.e. IDUA (NG_008103.1) exon 1-14, and IDS (NG_011900.2) exon 1-9. PCR products were purified and sequenced using a DNA sequencer. All amplified fragments flanking the exons were analyzed to identify variations. The resultant sequences were imported into Sequence Navigator software (Sequence Scanner Software 2, Applied Biosystems Inc., CA. USA) for alignment, editing, and mutation analysis [41–43].

## Results

A total of 294,196 and 153,032 newborn infants were screened by tandem mass spectrometry assay for MPS I and MPS II, respectively. Of these infants, 84 suspected cases were referred to MacKay Memorial Hospital for further confirmation due to values of either IDUA or IDS data being remarkably lower than the cut-off values, including eight for MPS I and 76 for MPS II.

### Urinary GAG quantification, 2D EP and disaccharide units of GAGs detected by LC-MS/MS assay

**Fig. 3:**
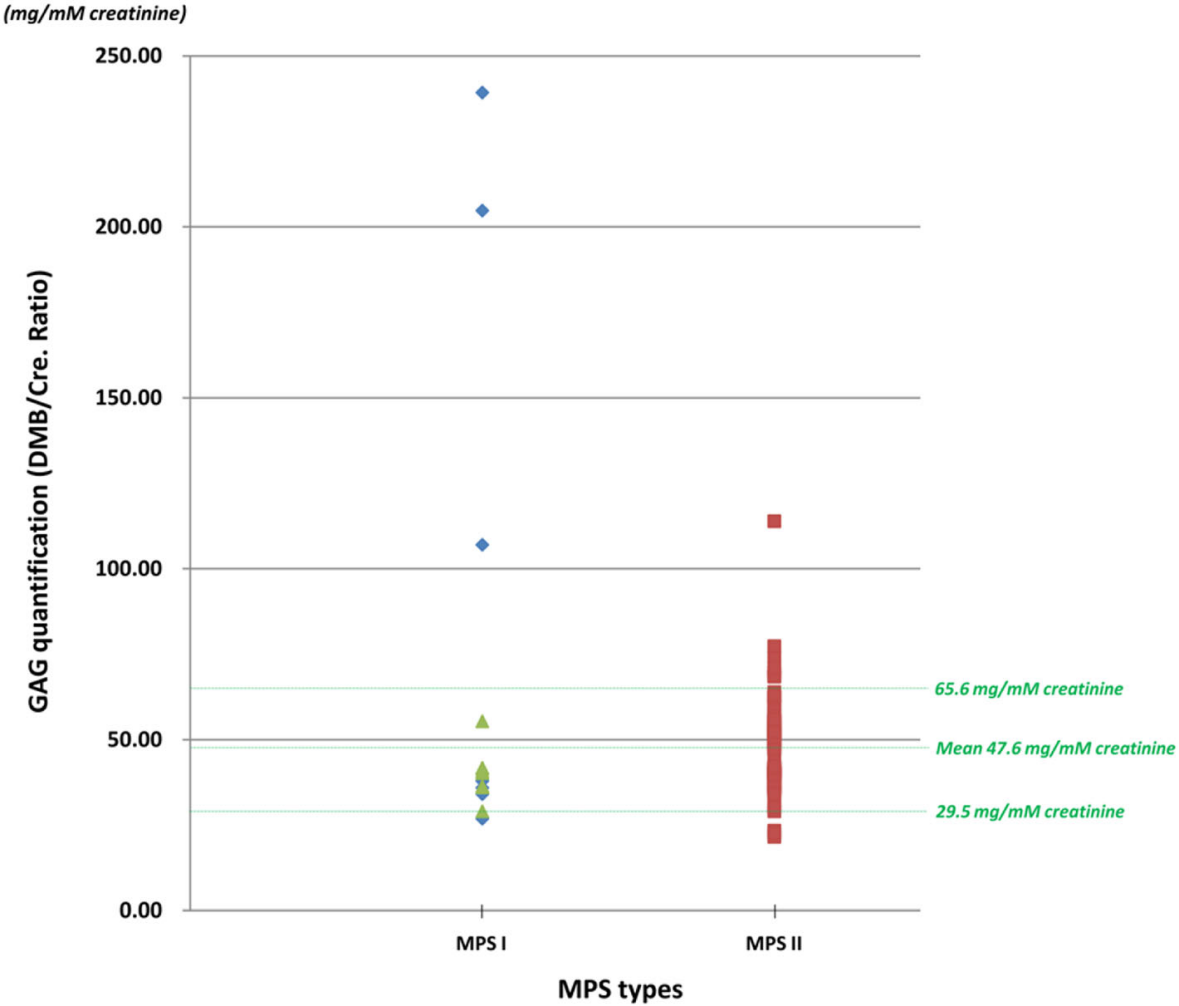
The DMB/CRE ratio of most of the referred cases in this study ranged from 29.5 to 65.6 mg/mM creatinine (mean ± SD = 47.6 ± 18.0 mg/mM creatinine) except for a few cases who were strongly suspected of having MPS I (*n* = 4) and MPS II (*n* = 7) with much higher values than the upper limit of the reference value

**Fig. 4:**
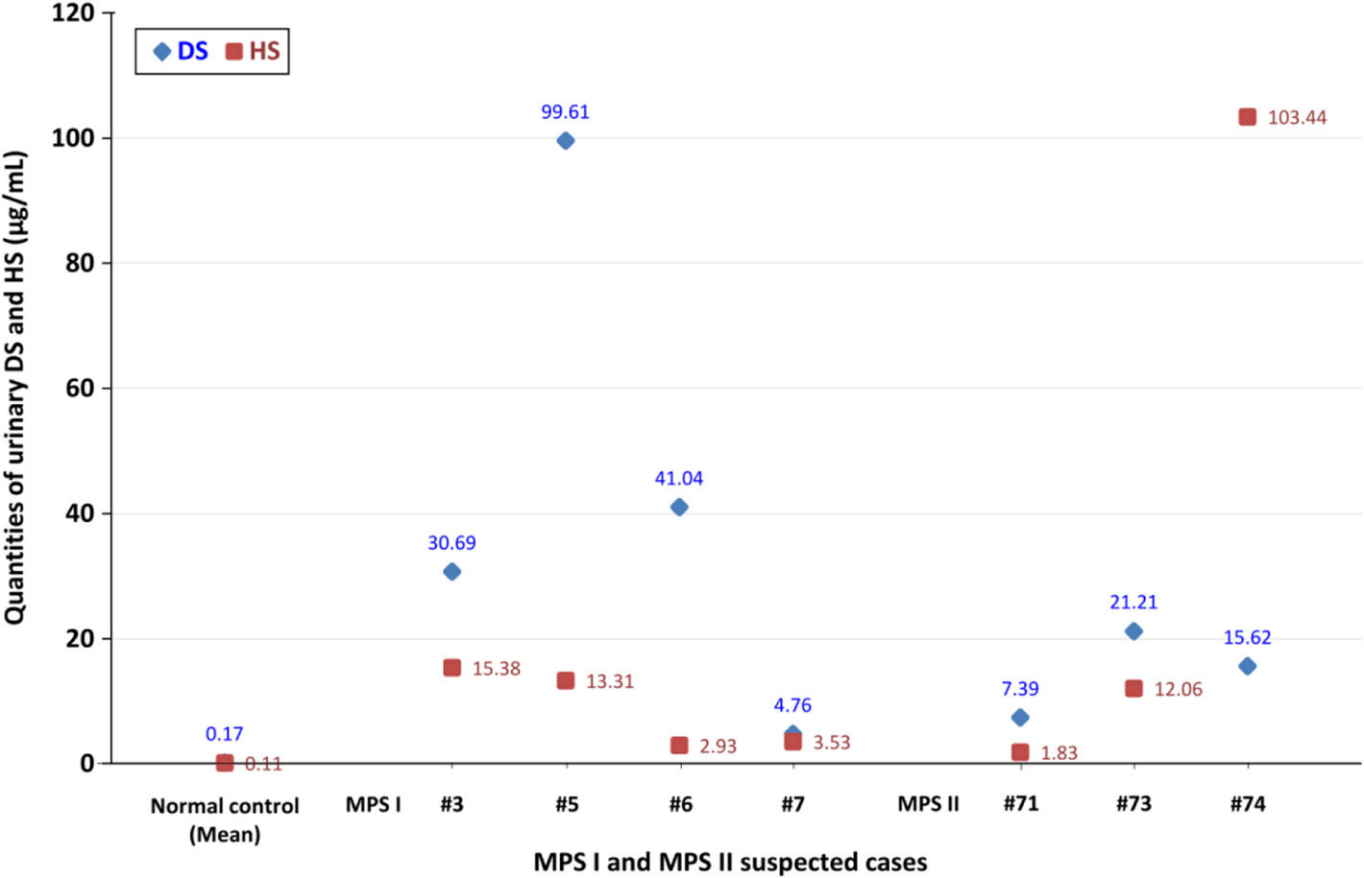
The average DS and HS values of the normal population were 0.17 (±0.22) and 0.11 (±0.21), respectively, whereas the DS and HS values were 4.76 to 99.61 and 2.93 to 15.38 μg/mL for the infants suspected of having MPS I, and 7.39 to 21.21 and 1.83 to 103.44 μg/mL for those suspected of having MPS II, respectively

The quantitative DMB method can give the ratio of excreted GAGs relative to creatinine, which is age dependent. A high ratio relative to age indicates the possibility of having MPS. According to our data, the urine creatinine level was proportional to age but inversely proportional to the DMB/CRE ratio. A higher DMB/CRE ratio was mostly noted in the very young group (< 2-year-old infants), whereas it was lower and nearly constant in the adult group. The normal reference values for the infants were 13.6-66.1 mg/mmol creatinine (< 6 months), and 0-55.2 mg/mmol creatinine (0.6-2 years). The DMB/CRE ratio of most of the referred cases in this study ranged from 29.5 to 65.6 mg/mM creatinine (mean ± SD = 47.6 ± 18.0 mg/mM creatinine) except for a few cases with highly suspected MPS I and MPS II that were much higher than the upper limit of the reference value (Fig. 3). Most of the referred cases had a normal 2D EP pattern, and only CS was present. With regards to the LC-MS/MS quantitative assay, seven highly suspected cases of MPS out of the 84 referred cases exhibited marked elevations of DS and HS (Fig. 4). The average DS and HS values of the normal population were 0.17 (±0.23) and 0.11 (±0.21), respectively, whereas the DS and HS values were 4.76-99.61 and 2.93-15.38 μg/mL in the cases with suspected MPS I, and 7.39-21.21 and 1.83-103.44 μg/mL in those suspected of having MPS II.

### Leukocyte IDUA and IDS enzymatic assay

After the urinary first-line biochemistry examinations, the possibility of MPS could be confidently ruled out in the referred cases with negative results. However, if the results were positive, i.e. increased levels of GAGs with DS + HS, or DS only, or HS only, the cases were highly suspected of having MPS. Leukocyte enzymatic assays were then performed in order to make a confirmative diagnosis of either MPS I or MPS II. In this study, both leukocyte IDUA and IDS enzymatic assays were analyzed including the eight cases with suspected MPS I and 76 with suspected MPS II. For the IDUA enzyme activity assay, four of the eight infants showed a marked reduction of 0.46 to 1.60 μmol/g protein/h (reference range: 4.87 to 54.70 μmol/g protein/h) that corresponded well with the results obtained from urinary first-line biochemistry examinations. The diagnoses of the four suspected cases were then proven. For the other four cases, three had significant reductions in IDUA enzyme activity (1.20, 1.50, and 2.06 μmol/g protein/h), and the other was normal (21.60 μmol/g protein/h); however these results did not correspond well with the results obtained from 2D EP and disaccharide LC-MS/MS assay. A further follow-up inspection and genotyping determination were thus required to exclude the possibility of MPS.

In the leukocyte IDS enzymatic assay of the 76 infants with suspected MPS II, 18 had normal values ranging from 12.80 to 40.80 μmol/g protein/4 h (reference value: 12.89-131.83 μmol/g protein/4 h) with negative urinary GAG examinations, 46 had marked reductions (3.86 ± 2.24 μmol/g protein/4 h) with negative urinary GAG assays, and the remaining three infants demonstrated definite deficiencies in IDS enzyme activities (0.20, 0.32, and 0.40 μmol/g protein/4 h) with positive urinary GAG first-line biochemistry examinations. Even though these three infants were confirmed as having MPS II by leukocyte enzymatic assay, no typical clinical presentations were noted. Molecular genetic analysis was then performed to determine the genotype.

### Molecular analysis of *IDUA* and *IDS* genes

**Fig. 5:**
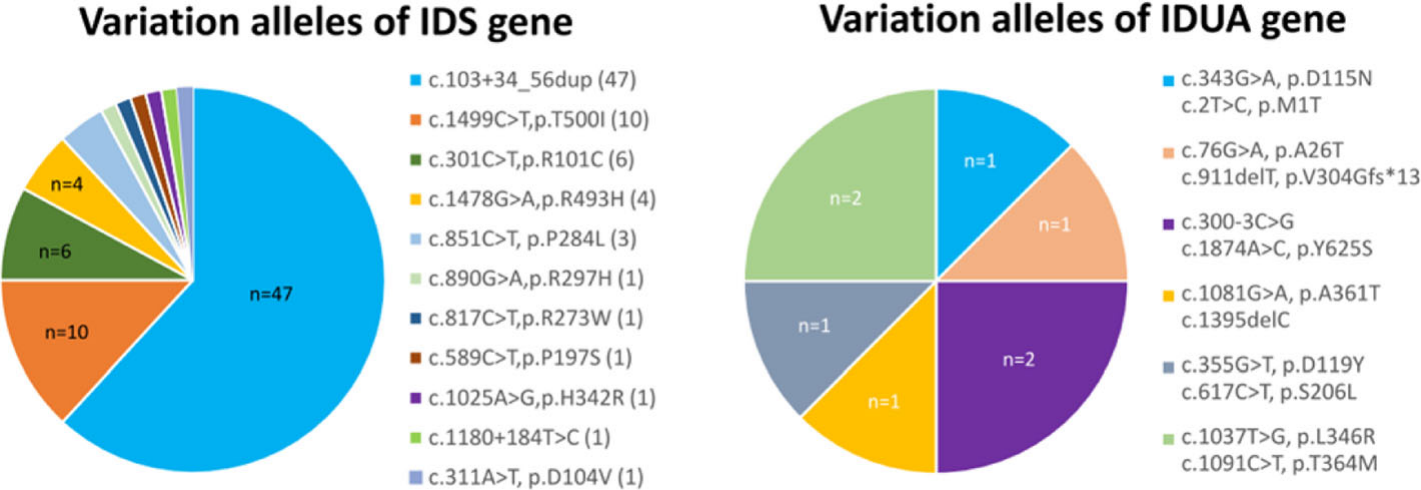
Molecular IDUA and IDS gene analyses. A total of 12 and 11 variation alleles of the IDUA and IDS genes were found, respectively. Of these alleles, four of 12 IDUA mutations (c.300-3C > G, c.1874A > C, c.1037 T > G, and c.1091C > T) were identified as being pathogenic and causing marked reductions in IDUA enzyme activity. In IDS gene molecular analysis, three mutation alleles (c.817C > T, c.1025A > G, and c.311A > T) were verified as being pathogenic genes that may cause deficiencies in IDS enzyme activity. The most common IDS variation allele found in this study was c.103 + 34_56dup, and the IDS enzyme activity appeared to be lower than 10 to 15% of normal when encoded with this variation allele

The results of molecular IDUA and IDS gene analysis showed 12 and 11 major variation alleles in the IDUA and IDS genes, respectively (Fig. 5a and b). Of these, four of 12 IDUA mutations (c.300-3C > G, c.1874A > C, c.1037 T > G, c.1091C > T) were identified as being pathogenic and caused marked reductions in IDUA enzyme activity in three infants [44, 45], two of whom were brother and sister, and two were twin sisters (Table 1). Most importantly, the results of the confirmatory diagnoses in either the leukocyte enzymatic assay or molecular gene analysis all corresponded well with the urinary GAG first-line biochemistry examinations. Other variations including c.343G > A, c.2 T > C [46]; c.355G > T [21], c.617C > T; and c.1081G > A [47], c.1395delC resulted in reductions in IDUA activity, and the urinary GAG first-line biochemistry examinations were all negative accordingly.

**Table 1:** Summary of the biochemical and molecular findings in the MPS I newborn screening test (IDUA gene)

Infant numbers	Sex	Ages (Ms) of the test	Ages (Yrs) at last follow up	Nucleotide ^(a)^	Protein ^(b)^	Exons	Known^Ref.^/ Novel	IDUA enzyme activity ^(c)^	GAG tests ^(d)^
1	Female			[c.2 T > C] + [c.343G > A]	[p.M1T] + [p.D115N]	1, 3	K [46]/N	1.20	Negative
2	Female			[c.76G > A] + [c.911delT]	[p.A26T] + [V304Gfs*13]	1, 7	N/N	21.60	Negative
3	Male^B^	1.1	1.8	[c.300-3C > G] + [1874A > C]	[−] + [p.Y625S]	3, 14	K [44]/N	1.60	Positive
4	Male			[c.355G > T] + [c.617C > T]	[p,D119Y] + [p.S206 L]	3, 6	K [21]/N	2.06	Negative
5	Female^T^	1.4	0.6	[c.1037 T > G] + [c.1091C > T]	[p.L346R] + [p.T364 M]	8, 8	K [44]/K [45]	0.81	Positive
6	Female^T^	1.4	0.6	[c.1037 T > G] + [c.1091C > T]	[p.L346R] + [p.T364 M]	8, 8	K [45]/K [45]	0.75	Positive
7	Female^S^	4.5	0.6	[c.300-3C > G] + [1874A > C]	[−] + [p.Y625S]	3, 14	K [44]/N	0.46	Positive
8	Male			[c.1081G > A] + [1395delC]	[p.A361T] + [−]	8, 9	K [47]/N	1.50	Negative

^E^ indicates Brother; ^S^ indicates Sister; ^T^ indicates Twin

^(a)^ Nucleotide variations [allele 1]+[allele 2]

^(b)^ Protein variations [allele 1]+[allele 2]

^(c)^ IDUA enzyme activity (Ref. 4.87 ~ 54.70 μmol/g protein/h)

^(d)^ GAG tests including GAG quantification (DMB/Cre ratio), 2-dimensional electrophoresis, and quantitative analyses of GAG-derived disaccharides (DS and HS) by tandem mass spectrometry assay

In IDS molecular analysis, three mutation alleles (c.817C > T, c.1025A > G, and c.311A > T) were recognized as being pathogenic and to have possibly caused marked reductions in IDS enzyme activity (Table 2). The other IDS variation alleles or polymorphisms including c.103 + 34_56dup, c.301C > T [48], c.1499C > T [49], c.1478G > A, c.589C > T, c.890G > A, c.851C > T [50], c.103 + 34_56dup + c.851C > T, and c.103 + 34_56dup + c.851C > T+ c.1180 + 184 T > C were proven to be non-pathogenic and to possibly lead to a pseudo-deficiency of IDS enzyme activity, and demonstrated negative results in the urinary GAG first-line biochemistry examinations. The possibility of having MPS II could confidently be ruled out in these 73 suspected patients.

**Table 2:** Summary of the biochemical and molecular findings in the MPS II newborn screening test (IDS gene)

Infant numbers	Sex	Ages (Ms) of the test	Ages (Yrs) at last follow up	Nucleotide Variation(s)	Protein Variation(s)	Exon	Known^Ref.^/ Novel	IDS enzyme activity ^a^	GAG tests ^b^
1-49	Male			[c.103 + 34_56dup]	[−]	1~ 2 (Intron-1)	Novel	3.86 ± 2.24	Negative
50-56	Male			[c.301C > T]	[p.R101C]	3	Known [48]	25.82 ± 9.04	Negative
57-66	Male			[c.1499C > T]	[p.T500I]	9	Known [49]	17.15 ± 3.69	Negative
67-70	Male			[c.1478G > A]	[p.R493H]	9	Novel	26.37 ± 10.98	Negative
71	Male	0.9	0.7	[c.817C > T]	[p.R273W]	6	Novel	0.20	Positive
72	Male			[c.589C > T]	[p.P197S]	5	Novel	7.80	Negative
73	Male	1.6	0.3	[c.1025A > G]	[p.H342R]	8	Novel	0.40	Positive
74	Male	0.9	0.3	[c.311A > T]	[p.D104V]	3	Novel	0.32	Positive
75	Male			[c.851C > T]	[p.P284L]	6	Known [50]	0.51	Negative
76	Male			[c.103 + 34_56dup] + [c.851C > T]	[−] + [p.P284L]	1~ 2 (Intron-1), 6	Novel/ Known [50]	1.29	Negative
77	Male			[c.103 + 34_56dup] + [c.851C > T] + [c.1180 + 184 T > C]	[−] + [p.P284L]	1~ 2 (Intron-1), 6 8~ 9 (Intron-8)	Novel/ Known [50]/Novel	4.50	Negative
78	Male			[c.890G > A]	[p.R297H]	7	Novel	9.20	Negative

^a^IDS enzyme activity (Ref. 12.89 ~ 131.83 μmol/g protein/4 h)

^b^GAG tests including GAG quantification (DMB/Cre ratio), 2-dimensional electrophoresis, and quantitative analyses of GAG-derived disaccharides (DS and HS) by tandem mass spectrometry assay

#### Variation allele c.103 + 34_56dup

**Fig. 6:**
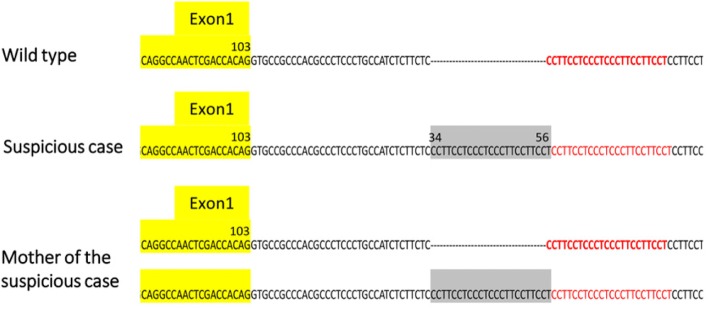
Variation allele, c.103 + 34_56dup, a novel IDS variation located between exon 1 and exon 2 (intron-1) which was down-stream of the 34 to 56 region had a repeat sequence, CCTTCCTCCCTCCCTTCCTTCCT

A total of 49 infants (Table 2, infant number 1-49) suspected of having MPS who underwent MPS NBS with tandem mass spectrometry assay were referred to MacKay Memorial Hospital for further confirmation (*n* = 49; 64.5% of 76 referred cases). A novel IDS variation allele, c.103 + 34_56dup, located between exon 1 and exon 2 (intron-1) down-stream of the 34 to 56 region had a repeat sequence, CCTTCCTCCCTCCCTTCCTTCCT (Fig. 6). This variation allele caused significant decreases in IDS enzyme activity in the initial and second DBS, ranging from 0.75 to 1.84 μmol/L/h which were significantly lower than the cut-off values of 6.5 μmol/L/h for the first test and 2.2 μmol/L/h for the second test. However, the leukocyte enzyme activity was low at an average 4.19 (±1.33) μmol/g protein/4 h and did not meet the diagnostic criteria of deficiency (less than 5% of the mean IDS enzyme activity in the normal population). Most notably, the results of urinary first-line biochemistry examinations were all negative, including the DMB/CRE ratio (46.74 ± 11.09 mg/mmol creatinine), 2-D EP (CS pattern only), and the disaccharides derived from GAGs as detected by LC-MS/MS (DS: 0.12 ± 0.14 μg/mL; and HS: 0.08 ± 0.10 μg/mL).

Another two suspected cases (infant number 76 and 77) who had the c.103 + 34_56dup variation allele also had the variation allele c.851C > T [p.P284L], and the variation allele c.851C > T [p.P284L] plus the variation allele c.1180 + 184 T > C, respectively. Reductions in leukocyte IDS enzyme activity were clearly found in both infants at 1.29 and 4.50 μmol/g protein/4 h, respectively, and both had negative urinary first-line biochemistry examinations.

#### Variation allele c.301C > T [p.R101C]

Six suspected infants (infant number 50-56) had the variation allele c.301C > T [p.R101C]. The IDS missense mutation was a rare polymorphism in our patients and did not affect IDS enzyme activity as verified by comparisons with the IDS activity in extracts of COS-7 cells expressing IDS from wild-type and mutant cDNA [48, 51]. The average IDS enzyme activity was 25.82 ± 9.04 μmol/g protein/4 h, and all of the results of urinary first-line biochemistry examinations were normal.

### Variation allele c.1499C > T [p.T500I]

Eight infants (infant number 57-66) had the variation allele c.1499C > T [p.T500I]. This allele could induce relatively lower levels of IDS enzyme activity in DBS compared with the normal level (2.49 ± 0.47 vs. < 2.2 μmol/L/h); however, the IDS enzyme activity in leukocytes seemed to be at the lower limit of normal, about 17.15 ± 3.69 μmol/g protein/4 h with negative results in the urinary first-line biochemistry examinations. The single-nucleotide polymorphism (SNP) database of The National Center for Biotechnology Information (NCBI) showed that the variation (c.1499C > T) was a SNP, and no previous study has reported that it is pathogenic to cause MPS II [*reference SNP: rs200120998*].

### Variation allele c.1478G > A [p.R493H]

Four infants (infant number 67-70) had the variation allele c.1478G > A [p.R493H], which caused relatively lower levels of IDS enzyme activity in DBS compared with the normal value (2.96 ± 0.71 vs. < 2.2 μmol/L/h); however, the leukocyte IDS enzymatic assay showed a normal result (26.37 ± 10.98 μmol/g protein/4 h) with negative results in the urinary first-line biochemistry examinations. No study has reported that the variation is pathogenic to cause MPS II.

### Variation alleles c.817C > T, c.1025A > G, and c.311A > T

Three infants (infant number 71, 73, and 74) with suspected MPS had either the variation allele c.817C > T [p.R273W], c.1025A > G [p.H342R], or c.311A > T [p.D104V]. These alleles could induce very low IDS enzyme activity in the DBS compared with normal values (0.39, 0.44, and 0.11 μmol/L/h, respectively vs. < 2.2 μmol/L/h). These novel IDS missense mutations were inherited from the infants’ mothers, and affected the synthesis of IDS protein leading to dramatic deficiencies in leukocyte IDS enzyme activity (0.20, 0.40, and 0.32 μmol/g protein/4 h, respectively). Urinary GAG biochemistry examinations were all positive, including distinct DS and HS patterns by 2D EP, critical elevations of DS and HS detected by tandem mass spectrometry assay, and high GAG/CRE ratios. The quantities of urinary DS and HS found in the suspected infants with variation alleles c.817C > T, c.1025A > G, and c.311A > T were 7.39, 21.21, 15.62 and 1.83, 12.06, 103.44 μg/mL, respectively (Fig. 4).

### Variation alleles c.890 G > A, c.589C > T, and c.851C > A

Two of three suspected infants (infant number 78 and 72) with the variation alleles c.890G > A and c.589C > T had moderate reductions in IDS enzyme activity of about 9.20 and 7.80 μmol/g protein/4 h, respectively. Both were novel missense mutations, and no previous study has reported that these variation alleles are pathogenic to cause MPS II. The other infant (infant number 75) had the c.851C > A variation allele and had a marked reduction in IDS enzyme activity (0.51 μmol/g protein/4 h). Even though the IDS enzyme activity was low, the urinary GAG analysis showed negative results, meaning that this case may have had a pseudo-deficiency of MPS II. This variation allele has been reported to be an attenuated phenotype [52]; however, Sawada et al. reported at the ACIMD conference in 2012 that this variation allele was not pathogenic to cause MPS II.

## Discussion

The MPS NBS program in Taiwan was initiated on August 1, 2015, and since then more than 294,196 and 153,032 infants have been screened for MPS I and MPS II, respectively. A total of 15 and 169 infants failed the first test for MPS I and MPS II in NBS, respectively (with cut-off values of < 3.0 for MPS I and < 6.5 μmol/L/h for MPS II), and were recalled for second DBS collection and second tests. Of these infants, eight and 76 failed the MPS I and II second test, respectively (with cut-off values of < 3.0 for MPS I and < 2.2 μmol/L/h for MPS II). The estimated recall rates were 0.005% for MPS I and 0.11% for MPS II. All of the 84 suspected infants were referred to MacKay Memorial Hospital for further confirmation, including regular physical examinations, urinary first-line biochemistry examinations, leukocyte enzymatic assay, and molecular genetic analysis. Four of the eight infants, including a brother and sister and twin sisters were confirmed to have MPS I, and three of the 79 cases with no related family members had MPS II. The positive predictive values of MPS I and II NBS were 26.7% (4/15) and 1.8% (3/169), respectively, which are relatively higher than that reported in Missouri (11%, with 4 pending) [27], and lower than that reported in Kentucky (50%). The Kentucky study showed that the number of cases requiring repeat analysis and a second-tier test such as GAG-derived disaccharides detected by tandem mass assay could drastically reduce false-positive outcomes [29]. In summary, the prevalence rates of MPS I and MPS II were 1.35 and 1.96 per 100,000 live births, respectively. No typical symptoms or signs of MPS have been noted in these infants to date, and detailed inspections every 6 months are still ongoing.

**Fig. 7:**
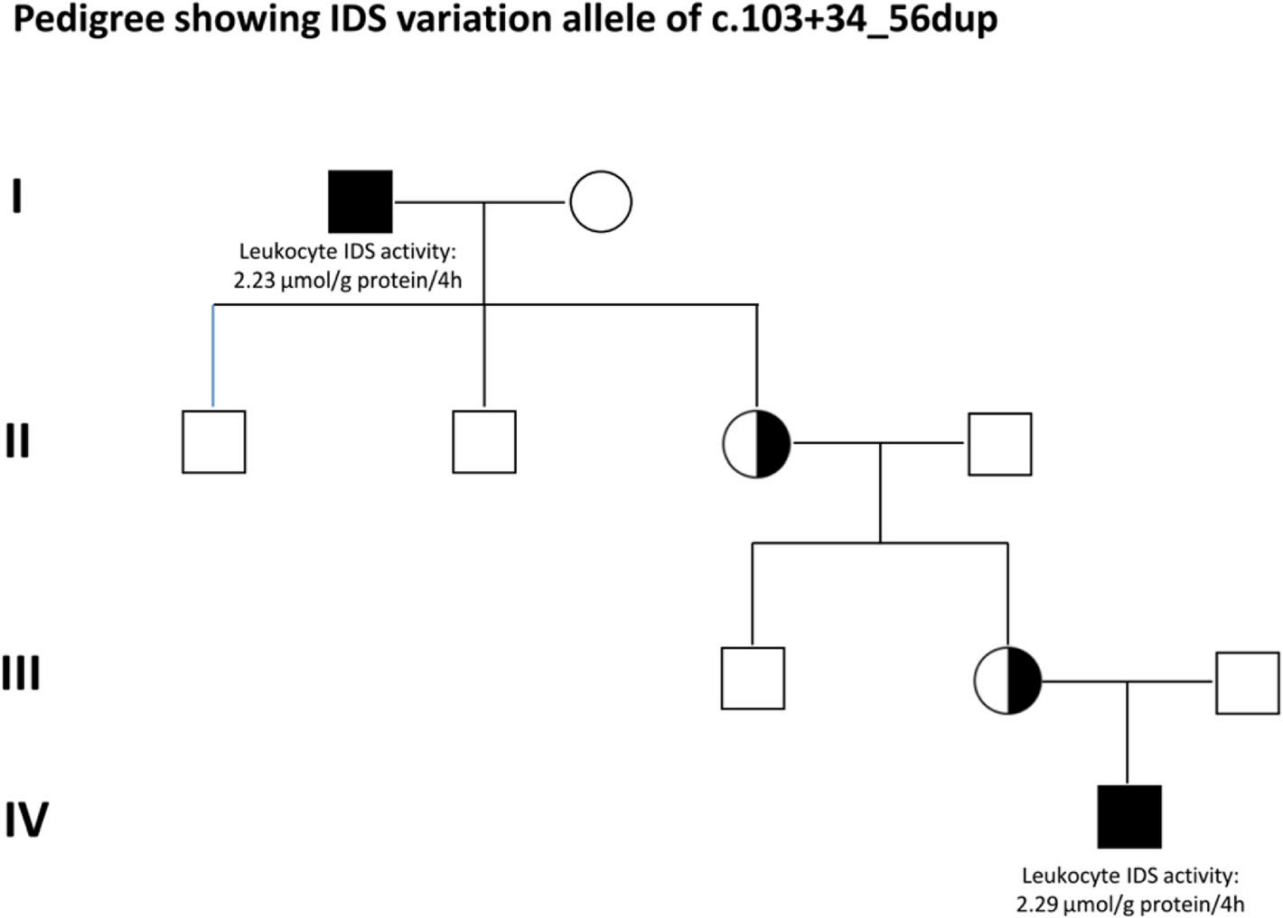
Four-generation pedigree showed that a great grandfather carried the same variation allele, c.103 + 34_56dup, but was healthy without any clinical presentations

In this study, a large number of suspected infants were identified with the variation allele c.103 + 34_56dup (*n* = 49, about 64.5%), and this variation caused a pseudo-deficiency of IDS enzyme activity. Because this variation allele is located between exon 1 and exon 2 (intron-1), the effectiveness of IDS enzyme activity was difficult to estimate according to the expression of IDS with c.103 + 34_56dup in COS-7 cells. In order to overcome this issue, RNA analysis was then performed. The cDNA sequencing analysis showed normal results, and there was no significant difference in RNA expression in real-time PCR analysis. In addition, we thoroughly investigated the male family members with the same variation via maternal inheritance from five different families. Most of the male maternal members including brothers and fathers did not have the same variation allele except for one family (Fig. 7). Four-generation pedigree showed that a 94-year-old great grandfather carried the same variation allele, and he was healthy without any clinical presentations.

Two suspected cases (infant number 66 and 67) were notable; one had the c.103 + 34_56dup variation allele accompanied with c.851C > T in exon 6, and the other had the variation allele c.851C > T plus c.1180 + 184 T > C. Sawada et al. reported that this variation may be a pseudogene of IDS and may cause structural modeling of the enzyme protein. A study of IDS with P284L confirmed that these amino acid substitutions are non-pathogenic. In addition, another study reported that the missense mutation c.851C > T caused an attenuated type of MPS II according to the exhibition of some clinical presentations [50].

Although the results from both MPS I and MPS II NBS were positive, the confirmation of MPS was difficult due to the asymptomatic presentations. In these conditions, performing expression experiments of IDUA or IDS novel mutations in COS-7 cells [48, 51], or gene related structural modeling experiments on the tertiary structure of IDUA and IDS enzymes [52, 53] is important to reveal whether or not the mutant is pathogenic to cause MPS. In addition, the clinical management of the suspected infants found in this study includes follow-up every 6 months, in particularly regular physician examinations for the earliest presenting symptoms such as otitis media, abdominal hernia, and facial features [54].

The gold standard for the diagnosis of MPS I and MPS II relies on the deficiency of IDUA and IDS activity in leukocytes, fibroblasts, or plasma with an unusual clinical presentation or a phenotype finding that does not match the results of GAG testing [55]. In the current study, several hemizygous non-pathogenic or pathogenic variants of the IDS gene were found with marked reductions in IDS enzyme activity; however, no typical clinical presentations were detected, and some of the infants had negative GAG test results. A definitive diagnosis of MPS II was thus difficult to make. Many studies have reported that the initial clinical signs and symptoms of MPS II emerge between the ages of 18 months and 4 years, depending on disease severity [55–57], and substantial delays have been reported in individuals with attenuated forms of MPS II. It is thus reasonable that the infants with suspected MPS II in this study had no clear clinical presentations due to their relatively young ages, ranging from 0.3 to 0.7 years at last follow-up. From the LC-MS/MS results of urinary GAG-derived disaccharides, the cases with either significant elevations of DS (MPS I #6) or HS (MPS II #74) should be more closely monitored in follow-up clinical inspections. This is because the gross accumulation of DS can affect and lead to soft tissue abnormalities such as cardiac valve, connective tissue, blood vessel, corneal, and skeletal deformities [58, 59], and HS may involve central nervous system (CNS) dysfunction [60, 61].

## Conclusion

MPS I and MPS II NBS programs have been available nationwide in Taiwan since August 2015, and these large-scale NBS programs are the first of their type. Since 2013, there have been three representative, large-scale NBS for lysosomal storage diseases including MPS I conducted in Missouri, Illinois, and Kentucky, USA, resulting in the development of an integrated screening algorithm and workflow. The entire diagnostic process including a first tier newborn screening test by tandem mass spectrometry assay and second tier confirmatory analysis are comprehensive, facile, and cost effective. Most importantly, it can provide the great benefits of health assurance with a very accurate diagnosis and allow for therapy in very early life, particularly when the irreversible MPS symptoms or clinical presentations have yet to occur. However, the positive predictive values of MPS I and II NBS are low, and the downstream emotional and financial cost of NBS tests should be carefully considered. Infants suspected of having MPS with a positive laboratory diagnosis but without any clinical presentations are required to undergo long-term regular physician examinations and laboratory tests in order to be able to give appropriate treatment in a timely fashion.

## Abbreviations

IDUA: α-Iduronidase
4MU: 4*-*Methylumbelliferone
CS: Chondroitin sulfate
DBS: Dried blood spot
DS: Dermatan sulfate
ERT: Enzyme replacement therapy
GAGs: Glycosaminoglycans
HS: Heparan sulfate
IDS: Iduronate-2-sulfatase
LSDs: Lysosomal storage disorders
MPS: Mucopolysaccharidosis

## References

[1] Besley GTN, Wraith JE (1997). Lysosomal disorders. Curr Paediatr.

[2] Wraith JE (1996). Mucopolysaccharidoses. Curr Paediatr.

[3] Neufeld E, Muenzer J. The mucopolysaccharidoses. In Valle D, Beaudet L, Vogelstein B, et al. The Online Metabolic and Molecular Bases of Inherited Disease. McGraw-Hill Global Education Holdings. 2001. 10.1036/ommbid.165.

[4] Lin HY, Chuang CK, Chen MR (2014). Natural history and clinical assessment of Taiwanese patients with mucopolysaccharidosis IVA. Orphanet J Rare Dis.

[5] Lin HY, Lin SP, Chuang CK (2009). Incidence of the mucopolysaccharidoses in Taiwan, 1984-2004. Am J Med Genet A.

[6] Nelson J (1997). Incidence of the mucopolysaccharidoses in Northern Ireland. Hum Genet.

[7] Baehner F, Schmiedeskamp C, Krummenauer F (2005). Cumulative incidence rates of the mucopolysaccharidoses in Germany. J Inherit Metab Dis.

[8] Jurecka A, Lugowska A, Golda A (2015). Prevalence rates of mucopolysaccharidoses in Poland. J Appl Genet.

[9] Patel P, Suzuki Y, Tanaka A (2014). Impact of enzyme replacement therapy and hematopoietic stem cell therapy on growth in patients with hunter syndrome. Mol Genet Metab Rep.

[10] Tanjuakio J, Suzuki Y, Patel P (2015). Activities of daily living in patients with hunter syndrome: impact of enzyme replacement therapy and hematopoietic stem cell transplantation. Mol Genet Metab.

[11] Wraith JE, Clarke LA, Beck M (2004). Enzyme replacement therapy for mucopolysaccharidosis I: a randomized, double-blinded, placebo-controlled, multinational study of recombinant human alpha-L-iduronidase (laronidase). J Pediatr.

[12] Tomatsu S, Alméciga-Díaz C, Barbosa H (2013). Therapies of mucopolysaccharidosis IVA (Morquio a syndrome). Expert Opin Orphan Drugs.

[13] Decker C, Yu ZF, Giugliani R (2010). Enzyme replacement therapy for mucopolysaccharidosis VI: growth and pubertal development in patients treated with recombinant human N-acetylgalactosamine 4-sulfatase. J Pediatr Rehabil Med.

[14] Brooks DA, Muller VJ, Hopwood JJ (2006). Stop-codon read-through for patients affected by a lysosomal storage disorder. Trends Mol Med.

[15] Hein LK, Bawden M, Muller VJ, Sillence D, Hopwood JJ, Brooks DA (2004). Alpha-L-iduronidase premature stop codons and potential read-through in mucopolysaccharidosis type I patients. J Mol Biol.

[16] Sands MS, Haskins ME (2008). CNS-directed gene therapy for lysosomal storage diseases. Acta Paediatr Suppl.

[17] Di Domenico C, Villani G, Di Napoli D (2009). Intracranial gene delivery of LV-NAGLU vector corrects neuropathology in murine MPS IIIB. Am J Med Genet A.

[18] Cearley CN, Wolfe JH (2007). A single injection of an adeno-associated virus vector into nuclei with divergent connections results in widespread vector distribution in the brain and global correction of a neurogenetic disease. J Neurosci.

[19] Muenzer J (2014). Early initiation of enzyme replacement therapy for the mucopolysaccharidoses. Mol Genet Metab.

[20] Al-Sannaa NA, Bay L, Barbouth DS (2015). Early treatment with laronidase improves clinical outcomes in patients with attenuated MPS I: a retrospective case series analysis of nine sibships. Orphanet J Rare Dis.

[21] Lin SP, Lin HY, Wang TJ (2013). A pilot newborn screening program for mucopolysaccharidosis type I in Taiwan. Orphanet J Rare Dis.

[22] Scott CR, Elliott S, Buroker N (2013). Identification of infants at risk for developing Fabry, Pompe, or mucopolysaccharidosis-I from newborn blood spots by tandem mass spectrometry. J Pediatr.

[23] Ruijter GJ, Goudriaan DA, Boer AM (2014). Newborn screening for hunter disease: a small-scale feasibility study. JIMD Rep.

[24] Van Diggelen OP, Zhao H, Kleijer WJ (1990). A fluorimetric enzyme assay for the diagnosis of Morquio disease type a. (MPS IV a). Clin Chim Acta.

[25] Tylki-Szymanska A, Czartoryska B, Bunge S (1998). Clinical, biochemical and molecular findings in a two-generation Morquio a family. Clin Genet.

[26] Camelier MV, Burin MG, De Mari J, Vieira TA, Marasca G, Giugliani R (2011). Practical and reliable enzyme test for the detection of mucopolysaccharidosis IVA (Morquio syndrome type a) in dried blood samples. Clin Chim Acta.

[27] Hopkins PV, Campbell C, Klug T (2015). Lysosomal storage disorder screening implementation: findings from the first six months of full population pilot testing in Missouri. J Pediatr.

[28] Burton BK, Charrow J, Hoganson GE (2017). Newborn screening for lysosomal storage disorders in Illinois: ihe initial 15-month experience. J Pediatr.

[29] Minter Baerg MM, Stoway SD, Hart J, and et al.. Precision newborn screening for lysosomal disorders. Genet Med. 2017. doi: 10.1038/gim.2017.194. 10.1038/gim.2017.194 29120458

[30] Kumar AB, Masi S, Ghomashchi F (2015). Tandem mass spectrometry has a larger analytical range than fluorescence assays of lysosomal enzymes: application to newborn screening and diagnosis of Mucopolysaccharidoses types II, IVA, and VI. Clin Chem.

[31] Elliott S, Buroker N, Cournoyer JJ, Potier AM, Trometer JD, Elbin C, Schermer MJ, Kantola J, Boyce A, Turecek F, Gelb MH, Scott CR (2016). Pilot study of newbornscreening for six lysosomal storage diseases using tandem mass spectrometry. Mol Genet Metab.

[32] Liao HC, Chiang CC, Niu DM, Wang CH, Kao SM, Tsai FJ, Huang YH, Liu HC, Huang CK, Gao HJ, Yang CF, Chan MJ, Lin WD, Chen YJ (2014). Detecting multiple lysosomal storage diseases by tandem mass spectrometry--a national newborn screening program in Taiwan. Clin Chim Acta.

[33] Chennamaneni NK, Kumar AB, Barcenas M, Spáčil Z, Scott CR, Tureček F, Gelb MH (2014). Improved reagents for newborn screening of mucopolysaccharidosis types I, II, and VI by tandem mass spectrometry. Anal Chem.

[34] Chuang CK, Lin SP, Chung SF (2001). Diagnostic screening for mucopolysaccharidoses by the dimethylmethylene blue method and two dimensional electrophoresis. Zhonghua Yi Xue Za Zhi (Taipei).

[35] Chuang CK, Lin SPL, Lee SJ, Wang TJ (2002). MPS screening methods, the berry spot and acid turbidity tests, cause a high incidence of false-negative results in sanfilippo and morquio syndromes. J Clin Lab Anal.

[36] Auray-Blais C, Bhérer P, Gagnon R, Young SP, Zhang HH, An Y, Clarke JT, Millington DS (2011). Efficient analysis of urinary glycosaminoglycans by LC-MS/MS in mucopolysaccharidoses type I, II and VI. Mol Genet Metab.

[37] Chuang CK, Lin HY, Wang TJ, Tsai CC, Liu HL, Lin SP (2014). A modified liquid chromatography/tandem mass spectrometry method for predominant disaccharide units of urinary glycosaminoglycans in patients with mucopolysaccharidoses. Orphanet J Rare Dis.

[38] Zhang H, Young SP, Auray-Blais C, Orchard PJ, Tolar J, Millington DS (2011). Analysis of glycosaminoglycans in cerebrospinal fluid from patients with mucopolysaccharidoses by isotope-dilution ultra-performance liquid chromatography-tandem mass spectrometry. Clin Chem.

[39] Hopwood JJ, Muller V, Smithson A, Baggett N (1979). A fluorometric assay using 4-methylumbelliferyl α-L-iduronide for the estimation of α-L-iduronidase activity and the detection of hurler and Scheie syndromes. Clin Chim Acta.

[40] Voznyi YV, Keulemans JL, van Diggelen OP (2001). A fluorimetric enzyme assay for the diagnosis of MPS II (hunter disease). J Inherit Metab Dis.

[41] Lee-Chen GJ, Lin SP, Chen IS, Chang JH, Yang CW, Chin YW (2002). Mucopolysaccharidosis type I: identification and characterization of mutations affecting alpha-L-iduronidase activity. J Formos Med Assoc.

[42] Chang JH, Lin SP, Lin SC, Tseng KL, Li CL, Chuang CK, Lee-Chen GJ (2005). Expression studies of mutations underlying Taiwanese hunter syndrome (mucopolysaccharidosis type II). Hum Genet.

[43] Lin SP, Chang JH, Lee-Chen GJ, Lin DS, Lin HY, Chuang CK (2006). Detection of hunter syndrome (mucopolysaccharidosis type II) in Taiwanese: biochemical and linkage studies of the iduronate-2-sulfatase gene defects in MPS II patients and carriers. Clin Chim Acta.

[44] Teng YN, Wang TR, Hwu WL, Lin SP, Lee-Chen GJ (2000). Identification and characterization of -3c–g acceptor splice site mutation in human a -L-iduronidase associated with mucopolysaccharidosis type IH:S. Clin Genet..

[45] Lee-Chen GJ, Wang TR. Mucopolysaccharidosis type I: identification of novel mutations that causeHurler/Scheie syndrome in Chinese families. J Med Genet. 1997;34(11):939–41. 10.1136/jmg.34.11.939 PMC1051126 9391892

[46] Wang X, Zhang W, Shi H, Qiu Z , Meng Y, Yao F, Wei M. Mucopolysaccharidosis I mutations in Chinese patients: identification of 27 novel mutations and 6 cases involving prenatal diagnosis. Clin Genet. 2012;46.81(5):443–52. 10.1111/j.1399-0004.2011.01680.x 21480867

[47] Chistiakov DA, Savost'anov KV, Kuzenkova LM, et al. Molecular characteristics of patients with glycosaminoglycan storage disorders in Russia. Clin Chim Acta. 2014;436:112–20. 10.1016/j.cca.2014.05.010 24875751

[48] Keeratichamroen S, Cairns JR, Wattanasirichaigoon D, et al. Molecular analysis of the iduronate-2-sulfatase gene in Thai patients with hunter syndrome. J Inherit Metab Dis. 2008;31(Suppl 2):S303–11. 10.1007/s10545-008-0876-z 18500569

[49] The single-nucleotide polymorphism (SNP) database from The National Center for Biotechnology Information (NCBI). Reference SNP: rs200120998.

[50] Kosuga M, Mashima R, Hirakiyama A, Fuji N, Kumagai T, Seo JH, Nikaido M, Saito S, Ohno K, Sakuraba H, Okuyama T. Molecular diagnosis of 65 families with mucopolysaccharidosis type II (huntersyndrome) characterized by 16 novel mutations in the IDS gene: genetic, pathological, and structural studies on iduronate-2-sulfatase. Mol Genet Metab. 2016;118(3):190–7. 10.1016/j.ymgme.2016.05.003 27246110

[51] Charoenwattanasatien R, Cairns JR, Keeratichamroen S, et al. Decreasing activity and altered protein processing of human iduronate-2-sulfatase mutations demonstrated by expression in COS7 cells. Biochem Genet. 2012;50(11-12):990–7. 10.1007/s10528-012-9538-9 22990955

[52] Uttarilli A, Ranganath P, Matta D, et al. Identification and characterization of 20 novel pathogenic variants in 60 unrelated Indian patients with mucopolysaccharidoses type I and type II. Clin Genet. 2016;90(6):496–508. 10.1111/cge.12795 27146977

[53] Saito S, Ohno K, Okuyama T, Sakuraba H. Structural basis of Mucopolysaccharidosis type II and construction of a database of mutant Iduronate 2-sulfatases. PLoS One. 2016;11(10):e0163964. 10.1371/journal.pone.0163964 PMC5047593 27695081

[54] Wraith JE, Beck M, Giugliani R, Clarke J, Martin R, Muenzer J. HOS Investigators. Initial report from the hunter outcome survey. Genet Med. 2008;10(7):508–16. 10.1097/gim.0b013e31817701e6 18580692

[55] Scarpa M, Almássy Z, Beck M, et al. Mucopolysaccharidosis type II: European recommendations for the diagnosis and multidisciplinary management of a rare disease. Orphanet J Rare Dis. 2011;6:72. 10.1186/1750-1172-6-72 PMC3223498 22059643

[56] Martin R, Beck M, Eng C, Giugliani R, Harmatz P, Munoz V, Muenzer J. Recognition and diagnosis of mucopolysaccharidosis II (hunter syndrome). Pediatrics. 2008;121(2):e377–86. 10.1542/peds.2007-1350 18245410

[57] Schwartz IV, Ribeiro MG, Mota JG, et al. A clinical study of 77 patients with mucopolysaccharidosis type II. Acta Paediatr. 2007;96(Suppl 455):63–70. 10.1111/j.1651-2227.2007.00212.x 17391446

[58] Golda A, Jurecka A, Tylki-S. Cardiovascular manifestations of mucopolysaccharidosis type VI (Maroteaux-Lamy syndrome). Int J Cardiol. 2012;158:6–11. 10.1016/j.ijcard.2011.06.097 21737154

[59] Harmatz P, Shediac R. Mucopolysaccharidosis VI: pathophysiology, diagnosis and treatment. Front Biosci (Landmark Ed). 2017;22:385–406. 10.2741/4490 27814620

[60] Tomatsu S, Gutierrez MA, Ishimaru T, et al. Heparan sulfate levels in mucopolysaccharidoses and mucolipidoses. J Inherit Metab Dis. 2005;28(5):743–57. 10.1007/s10545-005-0069-y 16151906

[61] Coutinho MF, Lacerda L, Alves S. Glycosaminoglycan storage disorders: a review. Biochem Res Int. 2012;2012:471325. 10.1155/2012/471325 PMC3195295 22013531

